# Bleeding Risk Profile in Patients on Oral Anticoagulation Undergoing Percutaneous Coronary Interventions: A Prospective 24 Months Cohort Study

**DOI:** 10.3389/fcvm.2021.589426

**Published:** 2021-09-30

**Authors:** Sara Schukraft, Tibor Huwyler, Cindy Ottiger-Mankaka, Sonja Lehmann, Ezia Cook, Daphné Doomun, Ianis Doomun, Jean-Jacques Goy, Jean-Christophe Stauffer, Mario Togni, Diego Arroyo, Serban Puricel, Stéphane Cook

**Affiliations:** Department of Cardiology, University and Hospital Fribourg, Fribourg, Switzerland

**Keywords:** high bleeding risk, ACR-HBR criteria, percutaneous coronary intervention, oral anticoagulation, triple antithrombotic therapy

## Abstract

**Background:** The Academic Research Consortium has identified a set of major and minor risk factors in order to standardize the definition of a high bleeding risk (ACR-HBR). Oral anticoagulation is a major criterion frequently observed.

**Aims:** The objective of this study is to quantify the risk of bleeding in patients on oral anticoagulation with at least one additional major ACR-HBR criteria in the Cardio-Fribourg Registry.

**Methods:** Between 2015 and 2017, consecutive patients undergoing percutaneous coronary intervention were prospectively included in the Cardio-Fribourg registry. The study population included patients with ongoing long-term oral anticoagulation (OAC) and planned to receive triple antithrombotic therapy. Patients were divided in two groups: patients on OAC with at least one additional major ACR-HBR criteria vs. patients on OAC without additional major ACR-HBR criteria. The primary endpoint was any bleeding during the 24-month follow-up. Secondary bleeding endpoint was defined as Bleeding Academic Research Classification (BARC) ≥3.

**Results:** Follow-up was completed in 142 patients at high bleeding risk on OAC, of which 33 (23%) had at least one additional major ACR-HBR criteria. The rate of the primary endpoint was 55% in patients on OAC with at least one additional ACR-HBR criteria compared with 14% in patients on OAC without additional ACR-HBR criteria (hazard ratio, 3.88; 95%CI, 1.85–8.14; *p* < 0.01). Patients with additional major ACR-HBR criteria also experienced significantly higher rates of BARC ≥ 3 bleedings (39% at 24 months).

**Conclusion:** The presence of at least one additional ACR-HBR criterion identifies patients on OAC who are at very high risk of bleeding after percutaneous coronary intervention.

## Introduction

Dual antiplatelet therapy (DAPT) is mandatory after percutaneous coronary intervention (PCI) (1). Of the patients undergoing PCI, 5–10% are on long-term oral anticoagulation (OAC) for either atrial fibrillation, venous thromboembolism, or mechanical valves (2, 3). The ensuing triple antithrombotic therapy (TAT) significantly increases the risk of bleeding after PCI (4, 5). Recently, an Academic Research Consortium (ARC) has identified a set of 12 risk factors in order to standardize the definition of a high risk of bleeding after PCI (6). The ARC definition of High Bleeding Risk (ARC-HBR) is dichotomous, as patients are considered to be at high bleeding risk if at least one major or two minor criteria are met. A major criterion for ARC-HBR is expected to predict an incidence of Bleeding Academic Research Consortium (BARC) type 3–5 bleeding of ≥4% at 1 year and/or an intracranial hemorrhage risk (ICH) of ≥1% at 1 year after PCI. However, the prevalence of individual criteria and their prognostic value for bleeding events after PCI have to be formally assessed.

Oral anticoagulation is a major criterion frequently observed in patients undergoing PCI. This study was conducted to (a) estimate the incidence of bleeding in patients under TAT after PCI, (b) assess whether the presence of at least one additional bleeding risk factor according to the ARC-HBR classification further increases bleeding risk in patients on TAT, and (c) measure the ischemic risk in HBR patients.

## Methods

### Study Population

The Percutaneous Coronary Intervention Registry FRIBOURG (CARDIO-FR) registry is a single-center prospective registry enrolling all consecutive patients undergoing PCI between May 2015 and May 2017 at the University & Hospital Fribourg (Fribourg, Switzerland). The exclusion criteria are the inability to provide written informed consent and the unwillingness to participate in clinical follow-up. The present analysis included patients with ongoing long-term OAC planned to receive DAPT.

### Study Procedures

The indication for PCI was based on established European guidelines (7). PCI was performed per standard techniques. The choice and duration of peri- and post-procedural antithrombotic regimen was left to the discretion of the operator.

### Data Collection

A prospective clinical follow-up by phone or clinic visit was completed for all patients up to 24 months. The study complied with the Declaration of Helsinki, was approved by the local ethics committee (003-REP-CER-FR), and is registered at clinicaltrials.gov (NCT04185285). Written and informed consent for prospective follow-up was obtained from every patient.

### Patient and Public Involvement Statement

Patients and the public were not involved in the design and conduct of this research.

### Clinical Endpoints

The primary endpoint was any bleeding during the 24-month follow-up. The secondary bleeding endpoint was defined as Bleeding Academic Research Classification (BARC) ≥3 (8), which corresponds to bleeding associated with a hemoglobin drop of >3 g/dl or more serious complications such as bleeding requiring surgery, coronary artery bypass graft (CABG)-related bleeding, intracranial bleeding, or blood transfusion. A hierarchical approach was used to describe the degree of severity of bleeding. The secondary ischemic endpoint was a patient-oriented composite endpoint (POCE) of all-cause death, myocardial infarction (MI), and any revascularization. Clinical events were adjudicated by an independent committee. Information about the antithrombotic regimens was collected at hospital discharge. All reported events and endpoints were reviewed and adjudicated.

### Statistical Analysis

Two groups were defined for the primary analysis: patients on OAC with additional major HBR criteria (OAC with additional ARC-HBR) vs. patients on OAC as the only major HBR criteria (OAC without additional ARC-HBR).

Categorical variables are reported as count and percentages; continuous variables are reported as mean ± SD or median (25–75% interquartile range). Normality was assessed by visual inspection of histograms and the computation of Q–Q plots. Continuous variables were compared using the Student's *t*-test or the Wilcoxon rank-sum test according to their distribution. Categorical variables were compared using chi-square or Fisher exact test as appropriate. Furthermore, clinical outcomes were reported as Kaplan-Meier failure estimations. Hazard ratios were derived from univariate Cox regression. To account for between-group differences in baseline characteristics and in prognostic variables with regard to the primary endpoint, we performed a Cox regression analysis. All relevant variables (age, diabetes mellitus, staged procedure, and treatment allocation) were forced into the model. Likewise, we performed a multivariable Cox regression to provide an adjusted hazard ratio for one of the bleeding secondary endpoint (major bleeding). Given the low number of events (*n* = 17), we performed a backward stepwise regression to identify the strongest predictors and finally retained two variables (diabetes mellitus and age) while forcing group membership into the model.

Secondary analysis included bleeding outcomes in relation to prescribed TAT duration by an individual patient-level analysis performed after cessation of TAT in each patient. Statistical analyses were performed with Stata version 14.0 (StataCorp LP, College Station, TX, USA).

## Results

### Patient and Procedural Characteristics

**Figure 1:**
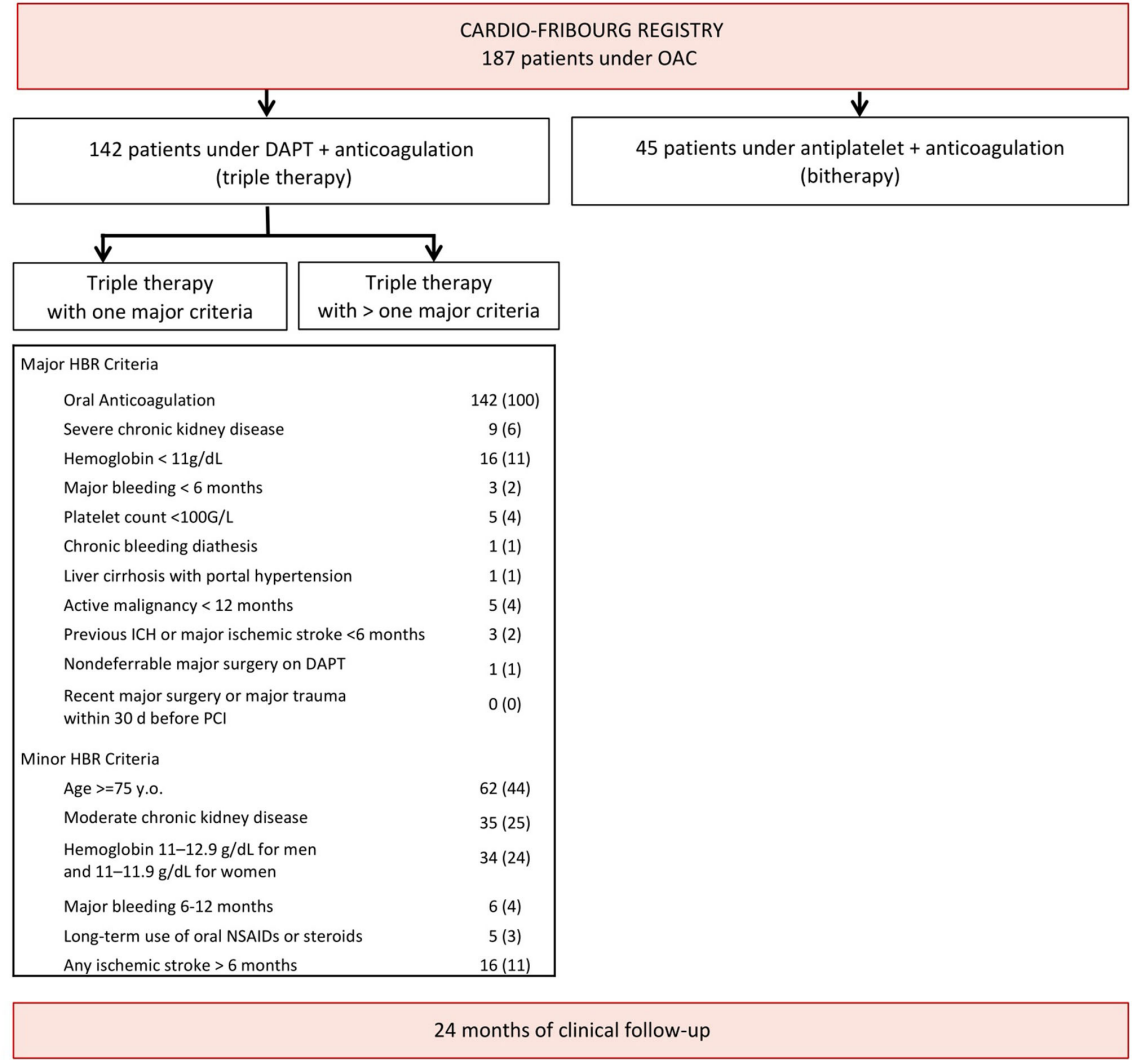
Study flow chart. DAPT, dual-antiplatelet therapy; HBR, high bleeding risk; ICH, intracerebral hemorrhage; NSAID, non-steroidal anti-inflammatory drugs; OAC, oral anticoagulation; PCI, percutaneous coronary intervention.

A total of 187 patients on OAC (17.3% of the cohort) underwent PCI during the inclusion period, of which 45 were excluded, as they received OAC and only single antiplatelet therapy (Figure 1). Of the 142 patients on OAC included in the analysis, 33 (23%) had at least one additional major ARC-HBR criteria. Table 1 summarizes baseline patient characteristics. Patients were older in the OAC with additional ARC-HBR group compared to the OAC– without additional ARC-HBR (77.89 ± 9.94 vs. 71.99 ± 10.32, *p* < 0.01).

**Table 1:** Baseline patient characteristics.

	**OAC+ additional** **ARC-HBR** **(***n =*** 33)**	**OAC– without** **additional** **ARC-HBR** **(***n =*** 109)**	***p***
Male	23 (70)	82 (75)	0.51
Age, years	77.89 ± 9.94	71.99 ± 10.32	<0.01
Hypertension	24 (73)	69 (63)	0.41
Diabetes mellitus	14 (42)	28 (26)	0.08
Non-insulin dependant	5 (16)	22 (20)	0.62
Current smoking	2 (6)	17 (16)	0.24
Dyslipidemia	17 (52)	43 (39)	0.23
Family history of CAD	3 (9)	19 (17)	0.41
Previous PCI	10 (30)	28 (26)	0.66
Previous CABG	5 (15)	16 (15)	1.00
Previous MI	6 (18)	17 (16)	0.79
**Clinical presentation**			
Unstable angina	3 (9)	5 (5)	0.39
NSTEMI	4 (12)	16 (15)	1.00
STEMI	3 (9)	23 (21)	1.13
Stable angina	4 (12)	30 (28)	0.10
Silent ischemia	4 (12)	7 (6)	0.28
Staged	15 (45)	28 (26)	0.05
LVEF, %	45 (32–63)	46 (31–65)	0.85

*Continuous variables are expressed as mean ± SD, categorical variables as counts and percentages*.

*ACS, acute coronary syndrome; CABG, Coronary Artery Bypass Graft Surgery; CAD, coronary artery disease; HBR, high bleeding risk; LAD, left anterior descending; LCX, left circumflex artery; LM, left main coronary artery; LVEF, Left ventricular ejection fraction; MI, myocardial infarction; NSTEMI, Non-ST elevation myocardial infarction; PCI, percutaneous coronary intervention; RCA, right coronary artery; STEMI, ST elevation myocardial infarction*.

Table 2 depicts the major and minor ARC-HBR criteria distribution. The median number of major HBR criteria in the OAC with at least one additional ARC-HBR group was 2 [2-3]. Lesion characteristics were similar in both groups (Supplementary Table 1).

**Table 2:** Patient characteristics according to major ARC-HBR criteria.

	**OAC+ additional** **ARC-HBR** **(***n =*** 33)**	**OAC– without** **additional** **ARC-HBR** **(***n =*** 109)**	***p***
**Major HBR criteria**			
Oral anticoagulation	33 (100)	109 (100)	1.00
Hemoglobin <11g/dL	16 (48)	0 (0)	<0.01
Severe chronic kidney disease (eGFR <30 ml/min)	9 (27)	0 (0)	<0.01
Previous ICH or major ischemic stroke <6 months	3 (9)	0 (0)	<0.01
Active malignancy <12 months or actively treated	5 (15)	0 (0)	<0.01
Platelet count <100 G/L	5 (15)	0 (0)	<0.01
Major bleeding <6 months	3 (9)	0 (0)	<0.01
Chronic bleeding diathesis	1 (3)	0 (0)	0.23
Liver cirrhosis with portal hypertension	1 (3)	0 (0)	0.23
Non-deferrable major surgery on DAPT	1 (3)	0 (0)	0.23
Recent major surgery or major trauma within 30 days before PCI	0 (0)	0 (0)	1.00
**Minor HBR criteria**			
Long-term use of oral NSAIDs or steroids	1 (3)	4 (4)	1.00
Age >75 years old	21 (64)	41 (38)	0.01
Moderate chronic kidney disease (eGFR 30–59 ml/min)	12 (36)	35 (32)	0.68
Hemoglobin 11–12.9 g/dl for men and 11–11.9 g/dl for women	8 (24)	26 (24)	1.00
Any ischemic stroke >6 months	4 (12)	12 (11)	1.00
Major bleeding 6–12 months	4 (12)	2 (2)	0.03

*Continuous variables are expressed as mean ± SD, categorical variables as counts and percentages*.

*DAPT, dual-antiplatelet therapy; HBR, high bleeding risk; ICH, intracerebral hemorrhage; OAC, oral anticoagulation; PCI, percutaneous coronary intervention*.

### Triple Antithrombotic Therapy

Vitamin K antagonist (VKA) were prescribed in 76 patients (54%), and 66 patients (46%) received a direct oral anticoagulant (DOAC). DAPT with aspirin and clopidogrel were prescribed in 123 patients (87%). The median duration for TAT was 1 month for both groups (*p* = 0.35). The indication for OAC was mainly atrial fibrillation in both groups (Table 3).

**Table 3:** Antithrombotic regimen after PCI.

	**OAC with** **additional ARC-HBR** **(***n =*** 33)**	**OAC without** **additional ARC-HBR** **(***n =*** 109)**	***p***
**Aspirin**	33 (100)	109 (100)	1.00
**P2Y12 inhibitor**			
Clopidogrel	29 (88)	94 (86)	1.00
Prasugrel	4 (12)	13 (12)	1.00
Ticagrelor	0 (0)	1 (1)	1.00
**Type of OAC**			
VKA	20 (61)	56 (51)	0.43
NOAC	13 (39)	53 (49)	0.43
**Triple therapy**	33 (100)	109 (100)	1.00
**Time of TAT, months**			
Mean ± SD	1.93 ± 1.73	2.68 ± 2.94	0.35
Median (IQR)	1 (1–3)	1 (1–3)	0.35
**Indication for OAC**			
AF, flutter	24 (73)	66 (61)	0.22
Venous thromboembolism/ Hypercoagulable syndrome	7 (21)	18 (17)	0.60
LV dysfunction, aneurysm or thrombus	2 (6)	21 (19)	0.10
Mechanical valve	0 (0)	4 (4)	0.57

*Categorical variables are reported as counts and percentages*.

*AF, atrial fibrillation; HBR, high bleeding risk; LV, Left ventricular; OAC, oral anticoagulation; NOAC, novel oral anticoagulant; TAT, triple antithrombotic therapy; VKA, vitamin K antagonist*.

### Primary Bleeding Endpoint

**Figure 2:**
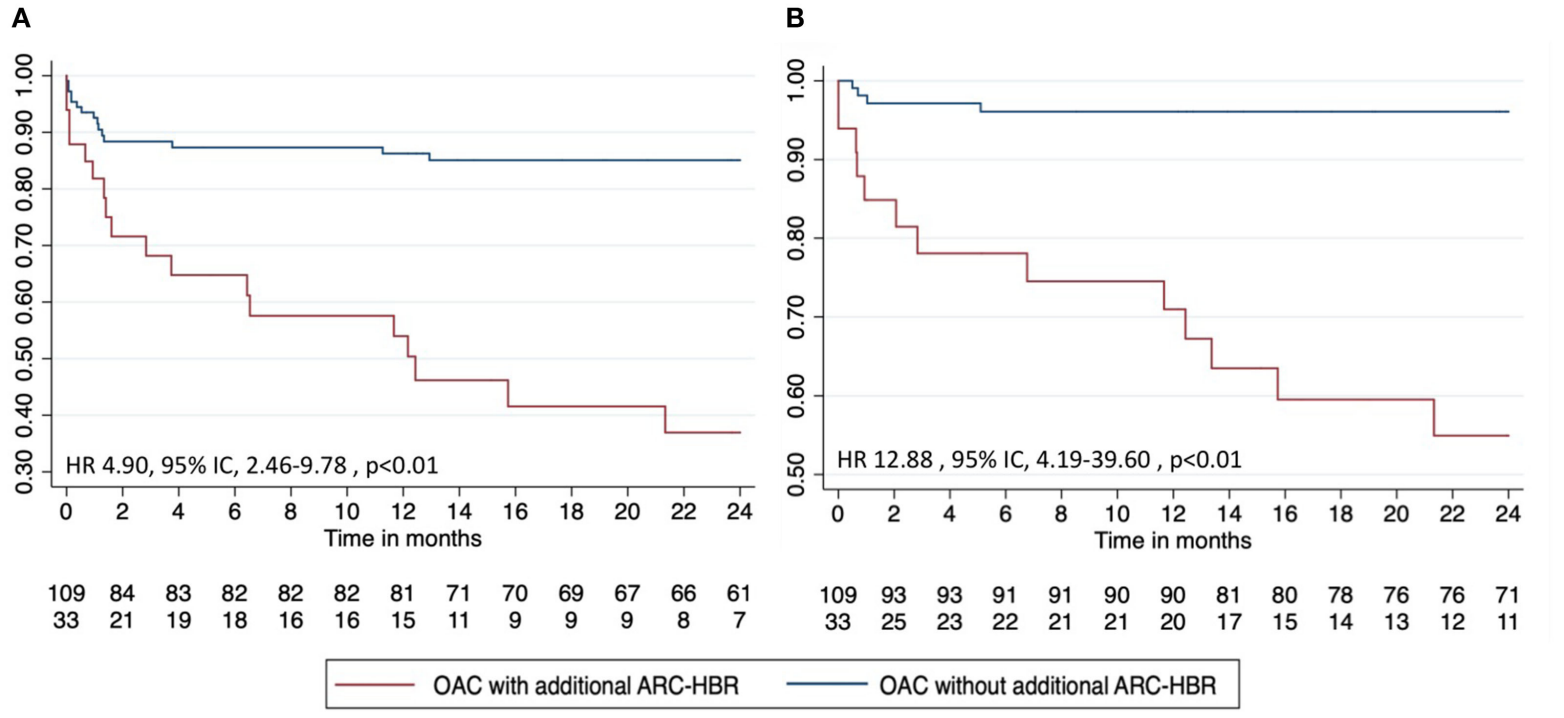
Bleeding-free survival according to major criteria for **(A)** any bleeding and **(B)** major bleeding. ARC, Academic Research Consortium High Bleeding Risk; HBR, high bleeding risk; OAC, oral anticoagulation; TAT, triple antithrombotic therapy.

**Figure 3:**
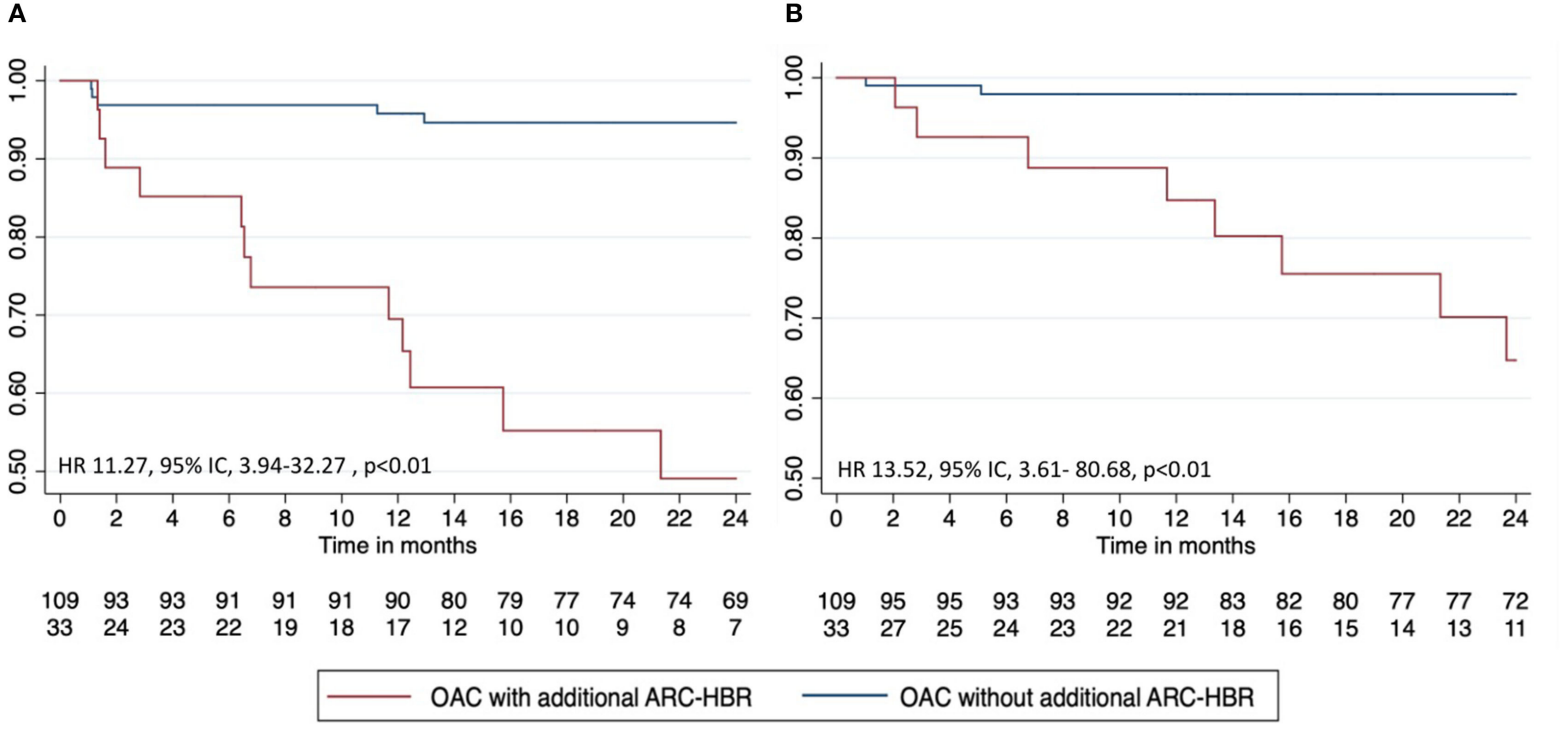
Landmark bleeding-free survival for **(A)** any bleeding and **(B)** major bleeding after TAT cessation. ARC, Academic Research Consortium High Bleeding Risk; HBR, high bleeding risk; OAC, oral anticoagulation; TAT, triple antithrombotic therapy.

At 24 months, the primary endpoint occurred in 55% (*n* = 18) of patients in the OAC with additional ARC-HBR group and in 14% (*n* = 15) in the OAC without additional ARC-HBR group (*p* < 0.01) (Figure 2, Table 4). After multivariate adjustment, the risk of bleeding remained significantly higher in patients with at least one additional major ARC-HBR criteria (hazard ratio, 3.88; 95%CI, 1.85–8.14; *p* < 0.01) (Table 5). The rate of major bleeding (BARC ≥ 3) occurred in 39% (*n* = 13) of patients in the OAC with additional ARC-HBR group and in 4% of patients (*n* = 4) in the OAC without additional ARC-HBR group (hazard ratio, 13.17; 95% CI, 4.02–43.17; *p* < 0.01) (Table 5). Interestingly, the difference persists after TAT cessation (Figure 3).

**Table 4:** Clinical outcome at 2-year follow-up according to major ARC-HBR criteria.

	**OAC with additional** **ARC-HBR**	**OAC without additional** **ACR-HBR**	**HR (95% CI)**	***p***
	**(***n =*** 33)**	**(***n =*** 109)**		
**Bleeding endpoint**				
Any bleeding	18 (55)	15 (14)	4.90 (2.46–9.78)	<0.01
BARC ≥ 3	13 (39)	4 (4)	12.88 (4.19–39.60)	<0.01
Any bleeding after TAT cessation	12 (36)	5 (5)	11.27 (3.94–32.27)	<0.01
BARC ≥ 3 after TAT cessation	8 (24)	2 (2)	13.52 (3.61–80.68)	<0.01
**Ischemic endpoint**				
POCE	4 (12)	16 (15)	0.91 (0.30–2.71)	0.86

*Values are n (%)*.

*BARC, Bleeding Academic Research Consortium; HBR, high bleeding risk; POCE, patient-oriented clinical end point defined as the composite of death, myocardial infarction, and any revascularization*.

**Table 5:** Adjusted clinical outcomes at 2 years of follow-up for primary and secondary bleeding endpoints.

	**Hazard ratio**	**95% CI**	***p***
**Any bleeding**			
OAC with additional ARC-HBR	3.88	1.85–8.14	<0.01
Age	1.05	1.00–1.09	0.03
Diabetes mellitus	0.79	0.37–1.70	0.55
Staged procedure	1.45	0.72–2.90	0.30
**Major bleeding**			
OAC with additional ARC-HBR	13.17	4.02–43.17	<0.01
Age	1.05	0.99–1.11	0.09
Diabetes mellitus	0.28	0.78–1.00	0.05

*ARC, Academic Research Consortium High Bleeding Risk; HBR, high bleeding risk; OAC, oral anticoagulation; STEMI, ST elevation myocardial infarction*.

### Secondary Ischemic Endpoints

**Figure 4:**
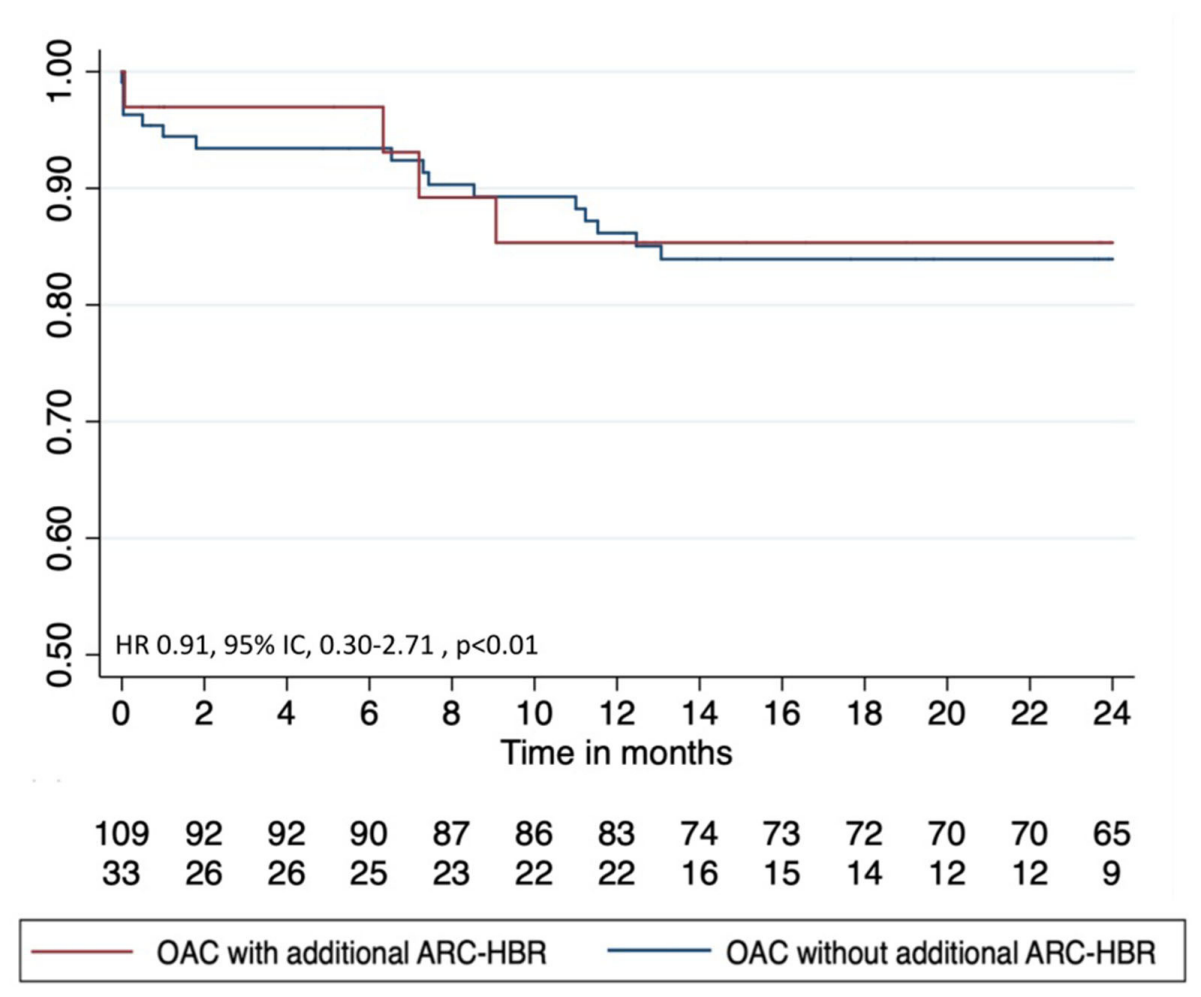
Twenty-four months survival free from the patient-oriented-composite endpoint. ARC, Academic Research Consortium High Bleeding Risk; HBR, high bleeding risk; OAC, oral anticoagulation.

The secondary combined ischemic endpoints at 24 months are provided in Table 4 and Figure 4. The incidence of POCE was 12% (*n* = 4) in the OAC with additional ARC-HBR group vs. 15% (*n* = 16) in the OAC without additional ARC-HBR group (hazard ratio, 0.91; 95% CI, 0.30–2.71; *p* = 0.86).

## Discussion

In this study, we observed that OAC is frequent in an all-comers registry of patients undergoing PCI. The main findings regarding OAC and bleeding risk are the following: (a) bleeding rates are high among patients on TAT; (b) in patients on TAT, the presence of at least one additional major ARC-HBR criteria is associated with a 3.9-fold increase in bleeding at 2-year clinical follow-up; (c) this difference persists after TAT cessation; and (d) there was no statistically significant difference in ischemic events in patients on OAC with additional HBR criteria compared to patients on OAC without additional ARC-HBR patients.

### Is the Bleeding Risk Associated With Triple Therapy?

We observed an overall incidence of any bleeding of 23% and major bleeding of 12% in 142 high-risk patients at 2 years. Several clinical trials have assessed bleeding events in TAT among participants with atrial fibrillation who underwent PCI. The incidence of bleeding in the major trials assessing TAT in patients with atrial fibrillation undergoing PCI was similarly high, although they varied depending on the type and duration of triple therapy, and patient characteristics. Overall bleeding rates range from 18 to 31% and severe bleeding of 1–6% in studies with 1 year of follow-up [the percentage of any bleeding outcome and severe bleeding were, respectively, 18 and 1% in PIONEER AF-PCI (9), 31 and 6% in WOEST (10), 20 and 6% in ENTRUST-AF (11), and 11 and 4% in WAR-STENT registry (12)]. The percentages of any bleeding outcome and severe bleeding were, respectively, 6–2% at 6 months in the AUGUSTUS trial (13), 10–4% at 9 months in the ISAR-TRIPLE (14), and 7–4% in RE-DUAL PCI (15) at 14 months. However, direct comparison of those trials is limited by the use of various trial-defined bleeding endpoint. Moreover, in all the trials comparing TAT vs. other regimens, patients with a multiple major HBR criteria were excluded.

### ARC-HBR Criteria and OAC

According to the ARC-HBR, the risk of a bleeding complication following PCI is defined by a number of major and minor criteria. This definition is dichotomous; in other words, a patient is declared as high bleeding risk or not. However, the interaction between each criterion is unknown, and thus, clinical validation is required. Natsuaki et al. (16) demonstrated, in a large Japanese cohort of 13,018 patients, an incremental increase in GUSTO moderate to severe bleeding events according to the presence or absence of major and minor criteria as defined by the ARC-HBR. The risk of bleeding varies between 4.2% in patients without HBR-criteria, 13.3% with one major HBR-criteria, 19.3% with two major HBR criteria, and 25.7% in patients with three or more major HBR criteria at 2 years. However, the use of GUSTO moderate/severe bleeding to define major bleeding and the higher risk of hemorrhage in Japanese patients (17) limits the comparison with other registries. Moreover, the population did not include patients under direct oral anticoagulants (DOAC). Recently, the ARC-HBR definition has been applied in a large cohort (18) of patients who have undergone PCI. The population under oral anticoagulation was equivalent to 18.5% with a cumulative incidence of the primary bleeding endpoint of 11.4% at 1 year of follow-up. When the oral anticoagulation criterion was taken in isolation, the cumulative incidence of the primary bleeding endpoint was 7.2%.

However, the definition of bleeding events reported by Cao et al. diverges from the BARC type 3–5 criteria.

The present study focuses on the risk of bleeding in patient under OAC as a sole major criterion and addresses the relevance of additional ARC-HBR criteria. Indeed, the incremental risk is magnified in our registry (4 vs. 39% cumulative incidence of BARC ≥3 bleeding at 2 years in patients with one vs. two or more major criteria, respectively). In addition, our analysis provides a glimpse into off-label strategies such as triple therapy with more potent P2Y12 inhibitors, as 13% of patients received a TAT with prasugrel or ticagrelor.

### Bleeding Events After TAT Cessation

As recently demonstrated in a large meta-analysis including most TAT trials with DOAC, TAT cessation and switch to double antithrombotic therapy (DAT) significantly reduced the risk of bleeding (19). However, and only when taking together larger trials, this comes at the expense of an increase in ischemic events particularly myocardial infarction and stent thrombosis. This may be partly explained by the fact that most patients received clopidogrel, including a subset of non-responders, with a low antiplatelet effect after Aspirin interruption. In line with this, we have demonstrated that TAT cessation does lower the risk of bleeding, but the difference between OAC with additional HBR and OAC without additional HBR patients persists throughout the 24 months follow-up.

### The Relationship Between Bleeding and Ischemic Events

Overall, ACR-HBR patients may be at increased ischemic risk, as some risk factors present in the ARC-HBR definition are also global ischemic factors such as advanced age, renal failure, and active malignancy. If some HBR criteria are also ischemic risks, with shorter antithrombotic therapy duration in some patients, the ischemic risk could be expected to increase. This was partly observed in the WOEST study, where TAT was associated with an increase in the combined ischemic events of death, MI, TVR, stroke, and stent thrombosis. It has been hypothesized that this may be due in part to the interruption of the TAT due to bleeding complications (20). In the 371,431 patients included in SWEDEHEART (21), the risk of bleeding was inversely correlated with ischemic events. As ischemic events are driven both by patient characteristics and procedural characteristics (lesion complexity, number of stents, stent size, total length, etc.), it is uncertain how this interacts with bleeding risk and HBR criteria. Recognizing HBR criteria may help to choose to simplify the procedure and thus limit the risk of ischemic events.

### Limitations

This study had several limitations. First, the number of patients was small, and the study was based on a single-center experience. The type and duration of triple therapy was not predefined but was individualized according to physician judgment; our findings, nonetheless, reflect real-world practice. Triple therapy duration was reported according to prescription at hospital discharge and was not consistently recorded. Possible triple therapy discontinuation has not been measured. Unmeasured confounders (e.g., INR levels) have not been determined.

## Conclusion

In a high-risk population with triple therapy and PCI, an incremental risk of major bleeding was detected with the addition of major-HBR criteria, up to 39% of major bleeding at 24 months. Patients with two or more ARC-HBR major criteria should be regarded as extremely high risk for major bleeding.

## Data Availability Statement

The raw data supporting the conclusions of this article will be made available by the authors, without undue reservation.

## Ethics Statement

The studies involving human participants were reviewed and approved by Local Ethics Committee of canton Vaud (CER-VD). The patients/participants provided their written informed consent to participate in this study.

## Author Contributions

All the authors have made substantial contributions to the conception and design of the study, the data acquisition, data analysis, and interpretation, the writing of the paper and critical review for important intellectual content, and the final approval of the version to be submitted.

## Funding

Data collection was supported by an unrestricted grant from the Fonds Scientifique Cardiovasculaire (Fribourg, Switzerland).

## Conflict of Interest

The authors declare that the research was conducted in the absence of any commercial or financial relationships that could be construed as a potential conflict of interest.

## Publisher's Note

All claims expressed in this article are solely those of the authors and do not necessarily represent those of their affiliated organizations, or those of the publisher, the editors and the reviewers. Any product that may be evaluated in this article, or claim that may be made by its manufacturer, is not guaranteed or endorsed by the publisher.

## Abbreviations

ARC-HBR: Academic Research Consortium for HBR
BARC: Bleeding Academic Research Consortium
DAPT: dual-antiplatelet therapy
HBR: high bleeding risk
MI: myocardial infarction
NOAC: novel oral anticoagulation
OAC: oral anticoagulation
POCE: patient-oriented cardiac events
PCI: percutaneous coronary intervention
TT: triple antithrombotic therapy
TVR: target-vessel revascularization
VKA: vitamin K antagonist.
