# Supplementary material for: Bleeding Risk Profile in Patients on Oral Anticoagulation Undergoing Percutaneous Coronary Interventions: A Prospective 24 Months Cohort Study
Source: Front Cardiovasc Med. 2021 Sep 30;8:589426. doi: 10.3389/fcvm.2021.589426 (PMC8514702; doi:10.3389/fcvm.2021.589426)
Supplement: Supplementary file 1 [file Table_1.DOCX]

**Supplementary Table 1. Baseline procedural characteristics**

|  | **OAC with additional ARC-HBR (n=33)** | **OAC without additional ARC-HBR (n=109)** | **p-value** |
| --- | --- | --- | --- |
| Number of vessel diseased per patient | 2.03±0.88 | 2.04±0.85 | 0.98 |
| Number vessels treated per patient | 1.12±0.33 | 1.09±0.29 | 0.62 |
| Number of lesions diseased per patient | 2.79±1.69 | 2.95±1.97 | 0.87 |
| Number lesions treated per patient | 1.61±0.83 | 1.53±0.73 | 0.77 |
| Number of stent per lesion | 1.18±0.43 | 1.18±0.43 | 0.91 |
| Arterial access site |  |  |  |
| Radial | 8 (24) | 29 (27) | 1.00 |
| Femoral | 25 (76) | 80 (73) | 1.00 |
| **Lesion level analysis** | **(n=51)** | **(n=163)** |  |
| Target artery |  |  |  |
| LM | 0 (0) | 4 (2) | 0.58 |
| LAD | 23 (45) | 76 (47) | 0.87 |
| LCX | 6 (12) | 29 (18) | 0.39 |
| RCA | 19 (37) | 48 (29) | 0.30 |
| Arterial graft | 0 | 0 | - |
| Vein graft | 3 (6) | 6 (4) | 0.45 |
| Lesion complexity |  |  |  |
| A | 7 (14) | 18 (11) | 0.62 |
| B1 | 11 (22) | 60 (37) | 0.06 |
| B2 | 14 (27) | 43 (26) | 0.86 |
| C | 9 (18) | 31 (19) | 1.00 |
| Baseline TIMI flow per lesion |  |  |  |
| TIMI 0 | 6 (12) | 30 (18) | 0.39 |
| TIMI 1 | 3 (6) | 6 (4) | 0.45 |
| TIMI 2 | 6 (12) | 15 (9) | 0.59 |
| TIMI 3 | 36 (71) | 112 (69) | 0.86 |
| TIMI flow post-intervention per lesion |  |  |  |
| TIMI 0 | 0 (0) | 0 (0) | - |
| TIMI 1 | 0 (0) | 0 (0) | - |
| TIMI 2 | 1 (2) | 4 (2) | 1.00 |
| TIMI 3 | 50 (98) | 159 (98) | 1.00 |
| Restenotic lesion | 3 (6) | 11 (7) | 1.00 |
| Chronic total occlusion | 1 (2) | 2 (1) | 0.56 |
| Maximum pressure per lesion, atm | 15.73±5.17 | 15.57±4.22 | 0.76 |
| Overlapping stents per lesion | 16 (31) | 55 (34) | 0.87 |
| Pre-dilatation per lesion | 40 (78) | 130 (80) | 0.84 |
| Post-dilatation per lesion | 18 (35) | 53 (33) | 0.73 |

Continuous variables are expressed as mean±SD, categorical variables as counts and percentages. BMS, bare-metal stent; DES, drug-eluting stent, LAD, left anterior descending; LCX, left circumflex artery; LM, left main coronary artery; RCA, right coronary artery; TIMI, thrombolysis in myocardial infarction.
